# Compromised N-Glycosylation Processing of Kv3.1b Correlates with Perturbed Motor Neuron Structure and Locomotor Activity

**DOI:** 10.3390/biology10060486

**Published:** 2021-05-30

**Authors:** Fadi A. Issa, M. Kristen Hall, Cody J. Hatchett, Douglas A. Weidner, Alexandria C. Fiorenza, Ruth A. Schwalbe

**Affiliations:** 1 Department of Biology, East Carolina University, Greenville, NC 27858, USA; issaf14@ecu.edu; 2 Department of Biochemistry and Molecular Biology, Brody School of Medicine, East Carolina University, Greenville, NC 27834, USA; hallma@ecu.edu (M.K.H.); hatchettc14@students.ecu.edu (C.J.H.); fiorenzaa16@students.ecu.edu (A.C.F.); 3 Department of Microbiology and Immunology, Brody School of Medicine, East Carolina University, Greenville, NC 27834, USA; weidnerd@ecu.edu

**Keywords:** glycans, motor neurons, Kv3 channels, neuronal development, motor activity, excitability, cell surface localization, neurodegenerative disorders, spinal cord, Kv3 channel gating

## Abstract

**Simple Summary:**

Modifications in the repertoire of N-glycans at the cell surface are associated with neurological complications. However, knowledge of specific N-glycans as to their impact on neuronal excitability is lacking. Our proposed studies will elucidate the roles of distinct N-glycan structures in modulating potassium channel function in highly repetitive firing neurons. This study is instrumental in understanding the development and progression of neurological diseases, thus, opening the door for potential therapeutic options.

**Abstract:**

Neurological difficulties commonly accompany individuals suffering from congenital disorders of glycosylation, resulting from defects in the N-glycosylation pathway. Vacant N-glycosylation sites (N220 and N229) of Kv3, voltage-gated K+ channels of high-firing neurons, deeply perturb channel activity in neuroblastoma (NB) cells. Here we examined neuron development, localization, and activity of Kv3 channels in wildtype AB zebrafish and CRISPR/Cas9 engineered NB cells, due to perturbations in N-glycosylation processing of Kv3.1b. We showed that caudal primary (CaP) motor neurons of zebrafish spinal cord transiently expressing fully glycosylated (WT) Kv3.1b have stereotypical morphology, while CaP neurons expressing partially glycosylated (N220Q) Kv3.1b showed severe maldevelopment with incomplete axonal branching and extension around the ventral musculature. Consequently, larvae expressing N220Q in CaP neurons had impaired swimming locomotor activity. We showed that replacement of complex N-glycans with oligomannose attached to Kv3.1b and at cell surface lessened Kv3.1b dispersal to outgrowths by altering the number, size, and density of Kv3.1b-containing particles in membranes of rat neuroblastoma cells. Opening and closing rates were slowed in Kv3 channels containing Kv3.1b with oligomannose, instead of complex N-glycans, which suggested a reduction in the intrinsic dynamics of the Kv3.1b α-subunit. Thus, N-glycosylation processing of Kv3.1b regulates neuronal development and excitability, thereby controlling motor activity.

## 1. Introduction

Folding, trafficking and delivery of membrane proteins during neuronal development and maintenance are tightly controlled processes and N-glycans of proteins are major contributors of these cellular processes [1,2]. Congenital disorders of glycosylation (CDG) emphasize the importance of N-glycan attachment and processing on neuronal development and maintenance, it frequently has a neurological component, such as motor skills [3,4]. The molecular mechanisms of how aberrant N-glycosylation processing of neuronal proteins lead to neurodegenerative diseases is poorly understood. N-Glycans are divided into three different types: oligomannose, hybrid and complex. *MGAT* genes encode for N-acetylglucosaminyltransferases (GnTs) which initiate branch points on the conserved pentasaccharide [5]. GnT-I catalyzes the conversion of oligomannose-type N-glycans to hybrid type, which in turn gives rise to complex type [5]. The importance of hybrid- and complex-type N-glycans has been demonstrated in mice, as knockout of GnT-I is embryonically lethal [1,2]. Knowledge of the impact of N-glycosylation processing of GnT-1 substrate would contribute to a fuller understanding of the role of this process on neuron structure and function.

Voltage-gated Kv3 channels have unique gating properties characterized by a depolarized activation range, and very fast activation and deactivation rates, and thereby promote sustained, high-frequency firing in neurons [6,7,8]. There are four members (Kv3.1–Kv3.4) of the Kv3 α subunit subfamily, each having two conserved N-glycosylation sites that are occupied in the brain and spinal cord [9]. Kv3.3 is heavily expressed in cerebellum and spinal cord motor neurons of zebrafish [10]. Further spinocerebellar ataxia type 13 mutations in the Kv3.3 channels disrupt voltage dependent gating, K^+^ current amplitude and neuronal excitability culminating in neuronal loss and locomotor deficits [10,11,12]. Thus, it is possible that abnormal N-glycosylation processing of Kv3 channels would impair neuronal development, excitability, and viability.

Recently, we showed that N-glycosylation processing is a mechanism for regulating Kv3.1b/a α subunit distribution in cell membranes and Kv3 channel activity in B35 neuroblastoma (NB) cells [13,14,15] and cultured rat adult cortico-hippocampal neurons [16]. Moreover, N-glycosylation occupancy and sialic acid residues of glycans are critical in modulating Kv1 channel function [17,18,19,20,21]. These observations suggest that unoccupied N-glycosylation sites and processing of N-glycans of Kv3.1b α subunit of Kv3 channels perturb ion channel distribution along the axon and thus influence neuronal voltage-dependent properties that regulate excitability and firing properties of developing neurons. We postulate that disruption of N-glycan processing is likely to compromise neuronal development, maturation, and viability, thus impairing motor activity.

Herein, we expressed fully (WT) or partially glycosylated (N220Q) Kv3.1 protein in zebrafish, *Danio rerio,* to determine their effects on development of spinal cord motor neurons that control zebrafish swimming behaviors. All spinal hemi-segments of zebrafish contain three well-defined primary motor neurons: Rostral (RoP), Medial (MiP) and Caudal primary (CaP) neurons [22,23]. Each neuron has distinct development and morphology, including non-overlapping innervation pattern of the post-synaptic musculature [24,25]. Further development of motor neurons to maturation occurs from 36 to 48 h post fertilization.

We hypothesized that occupancy of N-glycosylation sites of the Kv3.1b α-subunit and attachment of N-glycan types controls neuronal development and maintenance by altering the distribution of Kv3.1b in neuronal-derived cells and the activity of Kv3.1b-containing Kv3 channels. We report that transient expression of partially glycosylated Kv3.1b in motor neurons caused severe maldevelopment of CaP neurons, as is evident from lessened axonal length, branch numbers, and synaptic formation, while expression of fully glycosylated Kv3.1b showed normal development. Replacement of complex N-glycans with oligomannose in neuroblastoma cells decreased Kv3.1b distribution to outgrowths and altered size and density of Kv3.1b-containing complexes due to the type of N-glycan attached to Kv3.1b and those at the cell surface. Opening and closing rates of outward ionic currents were reduced for cells solely expressing oligomannose compared to those expressing complex. Finally, larvae from embryos expressing N220Q in CaP neurons displayed reduced swimming locomotor activity relative to those from embryos expressing EGFP or WT Kv3.1b in CaP neurons.

## 2. Materials and Methods

### 2.1. Cell Culture and Construction of NB_1 Cells with Knockout of GnT-I Stably Expressing Various Forms of Kv3.1b

Rat NB_1 clonal cell line (parental cell line), as well as NB_1_WT (Control_WT) and NB1_N220/229Q (Control_N220/229Q) cell lines expressing various forms of rat Kv3.1b were created and maintained as described [16]. Previously, we used CRISPR/Cas9 technology to silence the rat *Mgat1* [26] gene. Protocols to engineer NB_1(-*Mgat1*) cell lines which stably express EGFP tagged WT and N220/229Q Kv3.1b proteins, referred to as NB_1(-*Mgat1*)_WT and NB_1(-*Mgat1*)_N220/229Q, respectively, were like those for NB_1_WT(-*Mgat2*) and NB1_N220/229Q(-*Mgat2*) cell lines [16].

### 2.2. Zebrafish

All animal procedures were conducted as approved by the Institutional Animal Care & Use Committee (IACUC) of East Carolina University. Adult WT (AB) zebrafish (*Danio rerio*) were obtained from two independent laboratories, then propagated in labs at ECU. The *Pseudoloma*-free AB strain was purchased from Sinnhuber Aquatic Research Laboratory (SARL). This WT strain was employed for motor activity studies. Another WT (AB) strain was obtained from Zebrafish International Resource Center and used for confocal microscopy studies. Adult WT (AB) zebrafish were housed at the *Pseudoloma*-free lab or the other zebrafish lab at 28 °C on a light/dark cycle of 14 h on and 10 h off. Fish older than 96 h of age were fed every morning and afternoon. The *Pseudoloma*-free fish were kept on a high protein diet of Gemma Micro pellets (Skretting, Tooele, UT, USA) while the other group had dry pellet food and live brine shrimp. Males and females were bred to collect fertilized eggs for microinjections of the various plasmid constructs to express Kv3.1b protein in CaP neurons. Transient expression of rat glycosylated (WT), partially glycosylated (N220Q) and unglycosylated (N220/229Q) Kv3.1b proteins in CaP neurons was achieved by injecting recombinant pTol vectors (150–200 pg), encoding EGFP or one of the three Kv3.1b-EGFP fusion proteins, transposase (5–15 pg, Thermofisher, Scientific, Waltham, MA, USA) and a trace of phenol red into WT AB strain fertilized eggs at the 1–2 cell stage developmental level using a Pneumatic PicoPump PV 820 (World Precision Instruments, Sarasota, FL, USA). The embryos were dechorionated and then screened for EGFP fluorescence at ~36 hpf (Olympus BX51WI, Shinjuku City, Tokyo, Japan) and ~48 hpf (Olympus IX73) for confocal microscopy and motor activity studies, respectively.

### 2.3. Kv3.1b Recombinant Vectors

Expression of EGFP was targeted to the CaP primary neurons using a recombinant vector, which we refer to as pTol2_EGFP. In short, the pMiniTol2 vector (Addgene, Waterton, MA, USA) was engineered to contain the minimal promoter from the zebrafish *Gata2* gene, three copies of the 125 bp motor neuron enhancer from the mouse *Mnx1* (Hb9) gene, and the EGFP coding sequence [10,25,27,28]. Improved Restriction Digest Ligation (IRDL) [29] was employed to construct the pTol2_EGFP vector to encode cDNA of WT, N220Q and N220/229Q at the 5′ end of EGFP. In brief, oligonucleotide-directed PCR was used to add BamHI sites to both the 5′ and 3′ ends of Kv3.1b which had its stop codon removed. Also, 2 nucleotides were added to the 5′end of the 3′ BamHI site to generate a seven amino acid linker (QDPPVAT) which kept EGFP in frame with Kv3.1b. Once the Kv3.1b cDNA was constructed, it was then ligated into the BamHI digested pTol2_EGFP vector using the IRDL technique.

### 2.4. Confocal Microscopy

At 48 hpf, microinjected embryos expressing EGFP or EGFP tagged Kv3.1b proteins were anesthetized with 0.02% tricaine (MS-222) containing water and embedded onto a microscope slide in 1% low melt agarose. Confocal imaging was conducted using the Carl Zeiss LSM 800 Microscope using either 20 × 0.50 or 40 × 1.0 objectives water immersion lenses. All images were acquired at 1 µm optical sections to ensure high optical spatial resolution for 3D digital rendering of neuronal morphology. Confocal images were imported into Imaris software version 9.3 for digital rendering and 3D reconstruction (Bitplane, South Windsor, CT, USA). Analysis was restricted to neurons within somites 10–18 to minimize variations in neuron size and developmental stage.

Immunohistochemistry of WT AB embryos for detection of endogenous Kv3.1 or those heterologously expressing either EGFP alone or the Kv3.1b N220Q mutation were fixed in 4% paraformaldehyde at 4 °C for 24 h. Fixation was followed by two successive washes with PBS at room temperature for 10 min. Tissue was then dehydrated with increasing concentration of ethanol (10%, 20%, 50%, 70%, 90%, 100% × 2) for 5 min each. Dehydration was followed by an hour incubation in 100% methanol at −20 °C. Tissue was then incubated with a blocking buffer for 2–4 h 4 °C followed by incubation in primary antibodies overnight. Buffer solution contained (5% normal goat serum, 2% bovine serum albumin, 0.1% Triton X-100 in PBS). The tissue was then washed three times with PBST for 1 h, then incubated with the appropriate secondary antibodies. Antibody dilutions were as followed: Kv3.1b Rabbit primary (1:500) (Alomone Lab, JRS, IL, USA), Znp-1 mouse primary (1:1000) (Zebrafish International Resource Center, Eugene, OR, USA). Secondary Alexa Fluor 488 goat anti-rabbit (1:1000); secondary Alexa Fluor 555 goat anti-mouse (ThermoFisher Scientific, Waltham, MA, USA).

### 2.5. Motor Activity of Kv3.1b Expressing Fish

Pooled larvae expressing EGFP in motor neurons were maintained in 100 mm dish, containing egg water at 28 °C. After motor activity measurement for 5 dpf, the larvae were housed in 250 mL beaker filled with a mixture of egg water and system water at populations of 12 larvae or less. The ratio of egg water to system water was used as follows: 75:25, 5 DPF: 50:50, 6DPF; 25:75, 7 DPF; and 0:100, ≥8 dpf. For recording of motor activity, a fish was added to 11 mL egg water in a well of a 6-well dish and allowed to acclimate for 12 min on lab bench. Upon placement of dish into holding chamber in Zantiks MW apparatus (Zantiks, Cambridge, UK), a program was run to measure motor activity of larvae in pixels at 1 min intervals. The “locomotion _tracking” protocol from Zantiks MW acquisition menu, which involves a 60 s acclimation period and a 10 s target acquisition period, followed by the actual 5 min tracking procedure, was used to acquire spontaneous motor activity. The protocol was run in triplicate for each plate. After the procedure the fish were removed from the plate and transferred to a 250 mL beaker containing clean larvae water and returned to the incubator. This process was repeated at 7, 9 and 19 dpf.

### 2.6. Total Membrane Purification and Glycosidase Treatment

Total membranes were purified from cells, and glycosidase digestions of glycoproteins in total membranes were conducted as previously described [13]. Protein concentrations were established by a modified Lowry assay. Sample aliquots were denatured and reduced in SDS-PAGE sample buffer for western and lectin blotting while the remainder were stored at −20 or −80 °C and needed.

### 2.7. Western Blotting Assay

Membrane proteins were separated on 10% SDS-PAGE gels for 1.7 h at 20 mA, then transferred to PVDF membranes (Millipore, Burlington, MA, USA) for 2.5 h at 250 mA. Blotted membranes were incubated with mouse anti-Kv3.1 antibody (Neuromab, Davis, CA, USA) and secondary antibodies and band color was developed using NBT/BCIP.

### 2.8. TIRF Microscopy

An Apo 60X 1.45 objective attached to an Olympus IX-71 microscope (Olympus, Shinjuku City, Tokyo, Japan) with an ORCA R2 deep cooled mono CCD camera was used to capture images of EGFP tagged Kv3.1b proteins from the various cell lines as previously described [15]. The detection settings, exposure time and software used to control microscope and capture images were also maintained. The percent of fluorescence in outgrowth, as well as particle analysis parameters of outgrowths (projections from the cell body) and cell body, were determined using Image J software. Fluorescent particles remaining after background subtraction (5–10,000 pixels) were included in the analysis. To determine differences due to the type of N-glycan attached to the Kv3.1b protein, we calculated the ratio between the change of given parameter due to complex type of N-glycans ((Control_WT–Control_N220/229Q)/Control_N220/229Q) relative to the change of the same parameter due to oligomannose type of N-glycans ((NB_1(-*Mgat1b*)_WT–NB_1(-*Mgat1b*)_N220/229Q)/NB_1(-*Mgat1b*)_N220/229Q). As such, a value close to 1 indicates minimal, if any, changes due to the type of N-glycan attached to the Kv3.1b protein, while values greater or less than 1 denote changes due to the type of N-glycan of the glycosylated Kv3.1b protein.

### 2.9. Whole Cell Recordings and Analysis

Electrophysiological measurements from cell lines stably transfected with Kv3.1b proteins were conducted in a similar manner as previously described [13,16]. Based on current densities in non-transfected cell lines, the endogenous currents were small relative to the various Kv3.1b transfected NB cell lines, like those reported for the NB (aka B35) cell line [13,16]. As such, whole cell tracings were analyzed when membrane seal resistance was about 1 GΩ, and maximum current amplitude was ≥800 pA. Voltage clamp protocols were employed to examine activation and deactivation of outward ionic currents. Digitized whole cell current recordings from activation protocols were used to determine the potential at which the conductance was half maximal (V_0.5_), the conductance slope factor (dV) and rise times, and those from deactivation protocol were used to analyze deactivation time constants (tau_off_) as described [13,16].

### 2.10. Data Analysis

Analyzed confocal microscopy data of reconstructed neurons were exported into Excel, then graphing and statistical analysis was performed using GraphPad Prism software v.3.03. CLAMPFIT 10.3 software (Molecular Devices, San Jose, CA, USA) was used for whole cell current data acquired via CLAMPEX 9.0. Other displayed data used Origin 2018b (OriginLab Corporation, Northhampton, MA, USA) for graphics and statistical analysis. Adobe Photoshop was used for western and lectin blot images. Data are shown as the mean ± S.E. Statistical comparison of two groups were accomplished using Student’s t-test while more than two groups were done using one-way ANOVA with either Kruskal-Wallis or Bonferroni adjustments for confocal microscopy and all other studies, respectively. Statistical significance was considered at *p* < 0.05, unless indicated. 

## 3. Results

### 3.1. N-Glycosylation Processing of the Kv3.1b Protein Impacts Motor Neuron Development in Zebrafish

**Figure 1:**
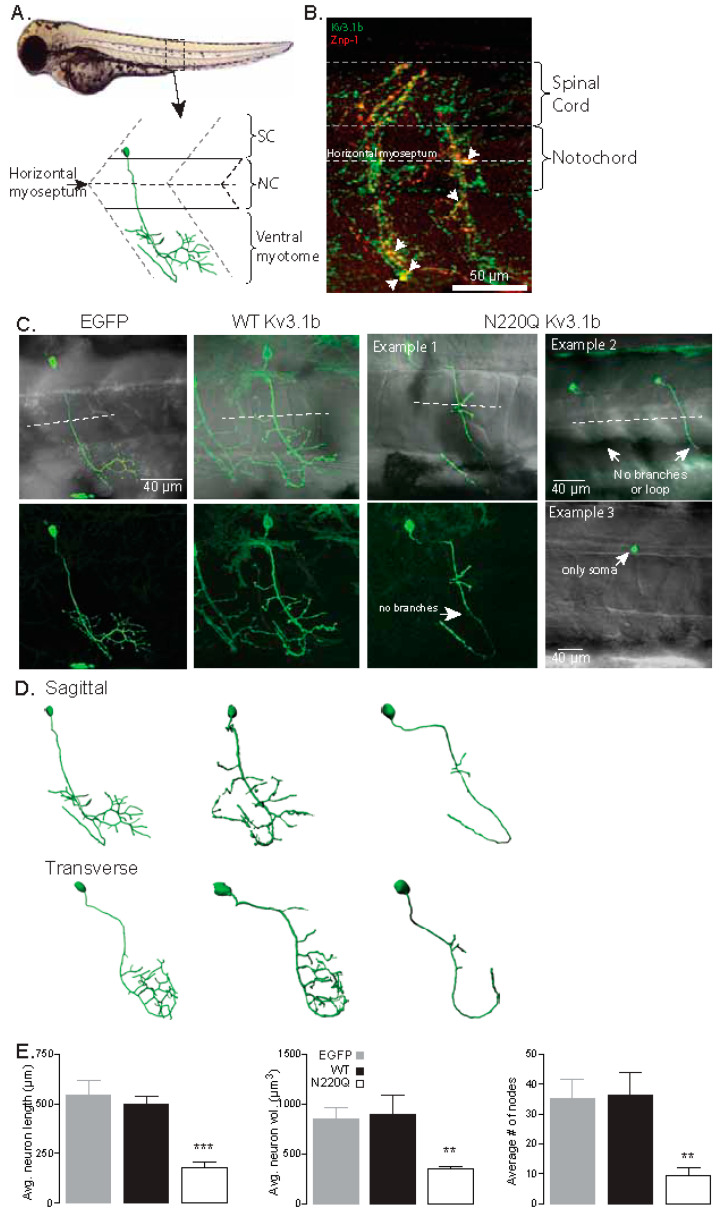
N-Glycosylation occupancy of Kv3.1b alters its spatial arrangement in motor neurons. (**A**) Schematic of a spinal hemi-segment illustrating the morphological innervation of the caudal primary motor neuron (CaP) of the ventral musculature. (**B**) A confocal projection (~40 µm stack) of one spinal hemi-segment illustrating the IHC co-staining of the Kv3.1b channel (Alexa Fluor 488, green) and Znp-1 (Alexa Fluor 555, red) in a CaP motor neuron. Arrowheads denote over-lapping expression of Kv3.1b and Znp-1. (**C**) Confocal projections of CaP neurons expressing EGFP, WT and N220Q Kv3.1b proteins. The bottom row shows confocal images, and top row images are overlaid with transmitted images to show the horizontal myoseptum (dashed line). (**D**) 3D digital rendering of the reconstructed neurons of images in (**C**) shown in both sagittal and transverse orientation. (**E**) Average axonal length, neuron axonal volume and number of nodes of CaP neurons expressing EGFP (*n* = 12), WT (*n* = 10) and N220Q (*n* = 11) Kv3.1b proteins. Graphs denote mean ± SEM and were compared by one-way ANOVA followed by Kruskal-Wallis adjustment (** *p* < 0.0005, *** *p* < 0.0001). See also Movies Figures S1–S3.

When one or both N-glycosylation sites in the Kv3.1b protein were vacant, the distribution of the Kv3.1b protein to outgrowths and Kv3 channel activity were adjusted compared to Kv3.1b protein with both sites occupied in NB cells [14] and primary neurons [16]. To elucidate the mechanism that lack of N-glycosylation occupancy of the Kv3.1b protein compromises the structure/function of motor neurons in vivo, we heterologously expressed fully (WT) and partially (N220Q) glycosylated forms of the Kv3.1b protein under the control of the motor neuron enhancer from the mouse Mnx1(Hb9) gene [28] in zebrafish embryos. Our focus was caudal primary (CaP) motor neurons, fast-spiking cells, of the spinal cord due to their well-defined morphology (Figure 1A), early time (24–52 hpf) of development and maturation, and lack of pigment to allow enhanced visualization and analysis. Further Kv3 channels containing the Kv3.3a α-subunit were shown to control CaP neuron excitability [10,25]. A representative confocal micrograph of an embryo (48 hpf) double labeled with anti-Kv3.1b and anti-Znp-1, a marker of axon and motor nerve terminals, antibodies revealed the expression of the endogenous Kv3.1b α-subunit in a highly branched axon extending deeply from the spinal cord across the horizontal myotome into the ventral musculature (Figure 1B). The extension of the process in this region was typical of CaP neurons, whereas the processes of the MiP (middle primary) and RoP (rostral primary) neurons do not enter the ventral myotome [22,23]. Taken together, the results indicated that Kv3.1b α-subunit is endogenously expressed in CaP neurons, and therefore, endogenous Kv3 channels are comprised of Kv3.1b, which controls neuronal excitability. 

Zebrafish embryos at the one-cell stage were microinjected with either recombinant pTol2 vectors expressing EGFP or one of the Kv3.1b (WT, N220Q or N220/229Q) proteins tagged with EGFP. The pTol2 vectors were designed for direct expression of candidate proteins to motor neurons [28]. At 36 hpf, embryos were screened for transient heterologous expression of EGFP in at least one of the CaP neurons. The mosaic Kv3.1b protein expression was typical and continued for up to 72 hpf. Most, if not all, of the embryos microinjected with unglycosylated Kv3.1b (N220/229Q) protein were aborted within 36 hpf or soon after (not shown). As such, the morphological development of CaP neurons expressing fully glycosylated Kv3.1b (WT) protein were compared to those expressing partially glycosylated Kv3.1b (N220Q). Confocal microscopy was employed to acquire images of CaP neurons expressing EGFP, WT and N220Q Kv3.1b proteins in live embryos from 48 to 54 hpf (Figure 1C). A confocal micrograph overlaid with a transmitted microscope image highlighted the spatial arrangement of EGFP expressed in CaP neurons relative to the spinal cord, notochord and ventral myotome (top left panel). EGFP expressing CaP neurons revealed the characteristic loop of CaP neuron under and around the ventral muscle, along with extensive branching around the ventral muscle. The high branching was clearly viewed in the confocal image (lower left panel). WT Kv3.1b expressing embryos also showed this stereotypical morphology and axonal trajectory (top middle panel). Of note, the characteristic loop with heavy branching of a CaP neuron in both adjacent hemi-segments was shown. In contrast, N220Q Kv3.1b-expressing CaP neurons displayed significant abnormal morphological development (top right panels). One neuron showed the process looping under and around the ventral muscle with minimal branching (Example 1). Two other CaP neurons had their axons crossing the horizontal myoseptum and extending into the ventral myotome region, but neither axon formed the characteristic loop or branches (Example 2). We also detected N220Q Kv3.1b solely in the soma (Example 3). Although it was unclear whether it was a CaP neuron, based on location, it was likely one of the three primary motor neurons. To characterize the effects of partially glycosylated (N220Q) K3.1b on CaP morphological development, we conducted 3D digital reconstruction of all imaged CaP neurons (Figure 1D). For lateral and cross-sectional visualization, CaP neurons were shown in the sagittal and transverse planes, respectively. This reconstruction provided a thorough examination of the neurons, regarding axonal length, number of branches and pathfinding errors (Figure 1E). Average neuron length (left panel) and volume (middle panel) were quite similar between EGFP- and WT Kv3.1b-expressing CaP neurons while these parameters were considerably reduced in the N220Q Kv3.1b-expressing neurons. Reductions in neuron length and volume were attributed to the lessened branching in CaP neurons expressing N220Q Kv3.1b protein as the average number of nodes were greatly decreased compared to those expressing EGFP or WT Kv3.1b proteins (right panel).

**Figure 2:**
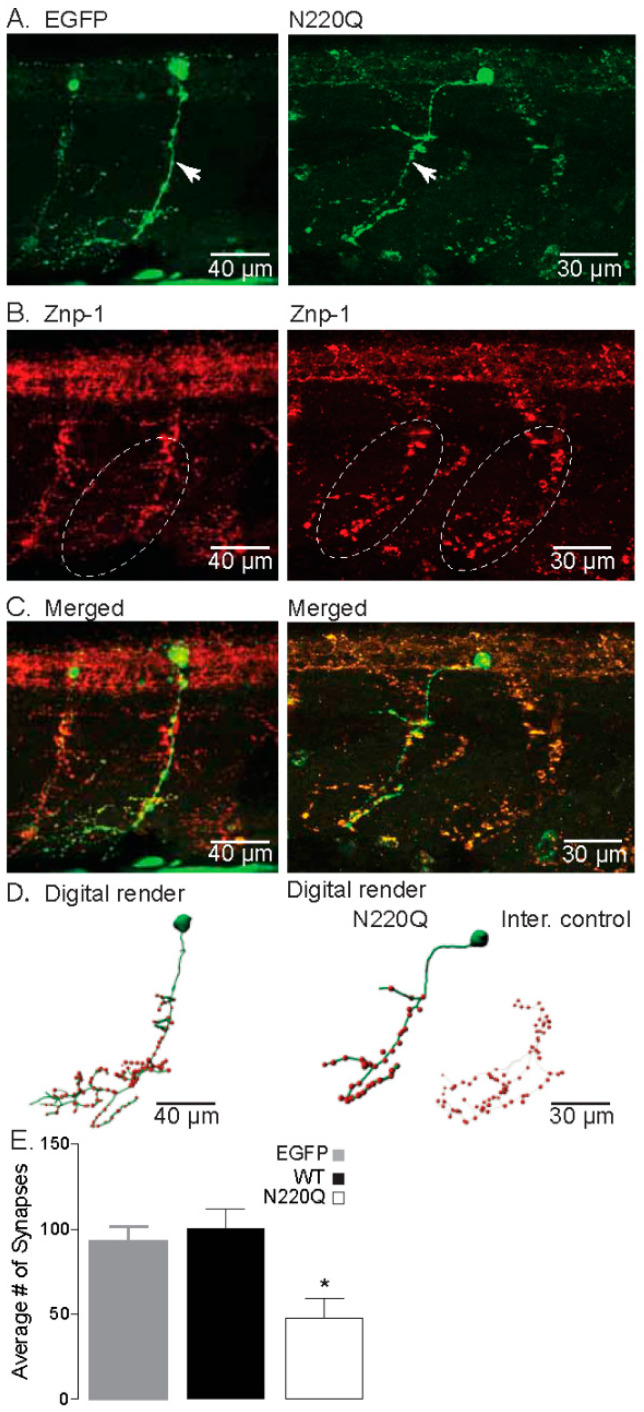
N-Glycosylation processing of the Kv3.1b protein impacts motor neuron development in zebrafish. (**A**) Confocal images of IHC staining of CaP neurons expressing either EGFP (**left**) or N220Q Kv3.1b (**right**) from 48 hpf embryos. Arrows denote main axonal branches. (**B**) Co-staining of Znp-1 highlights the extensive synapse points in the ventral myotome (dashed line oval). (**C**) Merged confocal projections from parts A and B, illustrating the lack of branches and lowered synapse numbers of CaP neurons expressing the N220Q Kv3.1b compared to EGFP alone or internal control. (**D**) Digital rendering of neuron morphology (green) and synaptic points (red) from part C, showing the extensive axonal branching of EGFP expressing neurons and internal control and high synaptic points compared to the CaP neuron expressing the N220Q Kv3.1b. (**E**) Average number of synapses of CaP neurons expressing EGFP (*n* = 10), WT (*n* = 8) and N220Q (*n* = 9) Kv3.1b proteins. Graphs denote mean ± SEM and were compared by Student’s t-test (* *p* < 0.0087). See also Movies Figures S4 and S5.

To determine whether the reduction in axonal branch number in N220Q Kv3.1b- expressing CaP neurons translated into a predicted decrease in synaptic formation, we co-labeled CaP neurons which expressed either EGFP or N220Q Kv3.1b with Znp-1, a specific presynaptic marker of synaptotagmin (Figure 2). As predicted, CaP neurons expressing EGFP protein showed extensive axonal branching and synaptic formation (Figure 2A–C, left panel). Conversely, CaP neurons expressing the EGFP tagged N220Q Kv3.1b protein showed reduced axonal branches that corresponded with decreased synapse Znp-1 staining (Figure 2A–C, right panel). These qualitative results were further verified using 3D digital rendering of the neurons (Figure 2D). The internal (inter) control was a CaP neuron that was imaged in a hemi-segment that did not express N220Q Kv3.1b protein from an embryo detected to express N220Q Kv3.1b. The mosaic expression of N220Q Kv3.1b protein allowed imaging of CaP neurons expressing N220Q Kv3.1b and those not expressing N220Q Kv3.1b (internal control) in the same embryo. In other words, an embryo microinjected with recombinant vector expressing N220Q Kv3.1b had both maldeveloped and stereotypical morphological CaP neurons. Quantification of the number of synapses was at least 2-fold higher in either EGFP- or WT Kv3.1b-expressing CaP neurons than in those expressing the N220Q Kv3.1b (Figure 2E). These results support that expression of partially glycosylated Kv3.1b hinders the development of CaP neurons.

### 3.2. N-Glycosylation Site Vacancy of Kv3.1b Hamper Motor Activity in Zebrafish Larvae

**Figure 3:**
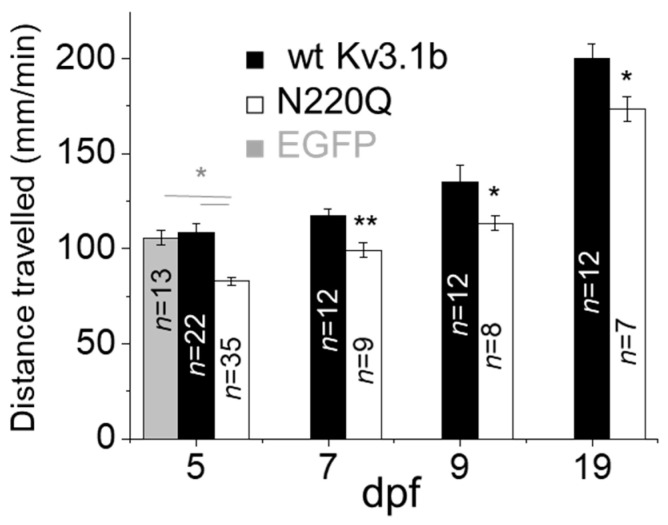
N-Glycosylation site vacancy of Kv3.1b retards motor activity in zebrafish. Average distance travelled per minute of larvae expressing EGFP, WT Kv3.1b or N220Q Kv3.1b in CaP neurons. Data are presented as the mean ± SEM and were compared by one-way ANOVA (* *p* < 0.01) and Student’s t-test (* *p* < 0.05, ** *p* < 0.01) for groups of more than two or two, respectively.

Primary motor neurons, such as CaP neurons, of zebrafish control rapid and large-amplitude movements in the ventral musculature, to coordinate locomotor activity [30,31]. Our aim was to demonstrate that defective CaP neuron development in embryos at 2 dpf was accompanied with reduced motor activity in larvae zebrafish. At 2 dpf, microinjected embryos were screened and grouped for heterologous expression of EGFP in at least one of the CaP neurons. Motor activity was measured at 5 dpf for microinjected embryos expressing EGFP, WT Kv3.1b or N220Q Kv3.1b in CaP neurons, and throughout the larvae period for the latter two groups (Figure 3). The average distance travelled per minute for larvae from EGFP and WT Kv3.1b groups were similar while those from the N220Q Kv3.1b group were lessened at 5 dpf. Further retarded motor activity was evident for the N220Q Kv3.1b larvae group compared to WT Kv3.1b larvae group up to 19 dpf. This small effect in motor activity was expected since recombinant pTol vector gives a mosaic expression pattern, such that the number of CaP neurons underdeveloped are at most 15% per embryo.

### 3.3. N-Glycosylation Mutant Neuronal-Derived Cell Model Expressing WT Kv3.1b with and without Oligomannose N-Glycans

**Figure 4:**
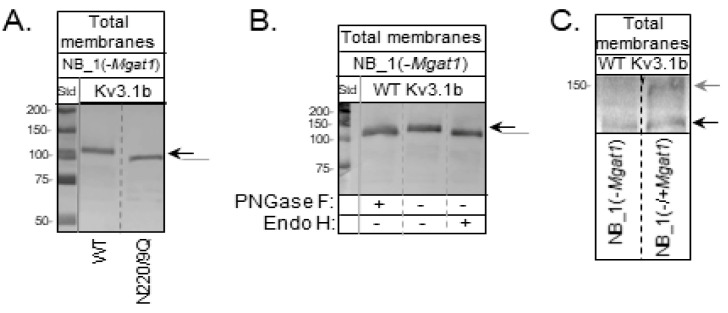
Immunoblot of Kv3.1b stable expressed in GnT-I knockout NB cells. (**A**) A Western blot of glycosylated (WT) and unglycosylated (N220/229Q) Kv3.1b proteins in total membranes from stably transfected NB_1(-*Mgat1*) cells. (**B**) A Western blot of total membrane proteins from NB_1(-*Mgat1*) cells stably expressing WT Kv3.1b digested (+) and undigested (−) with PNGase F and Endo H. (**C**) A Western blot of Kv3.1b from NB_1 (−*Mgat1*) cells stably expressing WT Kv3.1b compared to those cells transiently transfected with GnT-I. Molecular weight standards in kDa: 250; 150; 100; 75; 50; and 37 from top to bottom. Black and grey arrows denote Kv3.1b glycoprotein with oligomannose and complex types of N-glycans, respectively. The grey line signifies unglycosylated Kv3.1 protein. See also Figures S6–S8.

Since the current in vivo studies in zebrafish directly addressed our past in vitro studies [13,16], which suggested that N-glycosylation occupancy of the Kv3.1b α-subunit impacts neuronal development and function, we aimed to demonstrate that modifications in the type of N-glycans influences the spatial arrangement and channel activity of Kv3 channels containing Kv3.1b. This was addressed by stably expressing GFP tagged WT and N220/229Q Kv3.1b proteins in parental (NB_1) [16,32] and N-glycosylation mutant (NB_1(-*Mgat1*)) [26] cell lines. The earlier and later cell lines mainly express complex and oligomannose types of N-glycans, respectively. Further NB_1 cells produce WT Kv3.1b with complex type N-glycans occupying the two N-glycosylation sites, while the N220/229Q was unglycosylated [16,32]. A Western blot of the Kv3.1b protein in total membranes from NB_1(*-Mgat1*)_WT or NB_1(*-Mgat1*)_N220/229Q cell lines showed that glycosylated Kv3.1b protein migrated slightly slower than unglycosylated Kv3.1b (Figure 4A). These migration patterns were like those previously shown for Kv3.1b with oligomannose type N-glycans and the unglycosylated counterpart in CHO cells [33,34]. Further WT Kv3.1b glycoprotein migrated to a similar position as N220/229Q Kv3.1b protein upon treatment with either PNGase F (removes all types of N-glycans) or Endo H (removes N-glycans of oligomannose type and some hybrid type), indicating the removal of N-glycans attached to the WT Kv3.1b glycoprotein (Figure 4B). When the NB_1(*-Mgat1*)_WT cell line was transiently transfected with the mouse *Mgat1* gene, two immunobands were observed (Figure 4C). The faster immunoband corresponded with the WT Kv3.1b glycoprotein containing oligomannose type N-glycans while the slower migrating immunoband was like that observed for the WT Kv3.1b glycoprotein expressed in NB_1 cell line which has complex type N-glycans [33,34]. These results indicated that the NB_1(*-Mgat1*)_WT cell line could be rescued since at least some the oligomannose type of N-glycan attached to the Kv3.1b glycoprotein were converted to complex type. Our results support that the WT Kv3.1b protein has oligomannose N-glycans while the N220/229Q Kv3.1b protein lacked N-glycans in the NB_1(-*Mgat1*) cells. 

### 3.4. N-Glycosylation Processing Modified the Spatial Arrangement of Kv3.1b Protein-Containing Particles in Subdomains of the Cell Membrane

**Figure 5:**
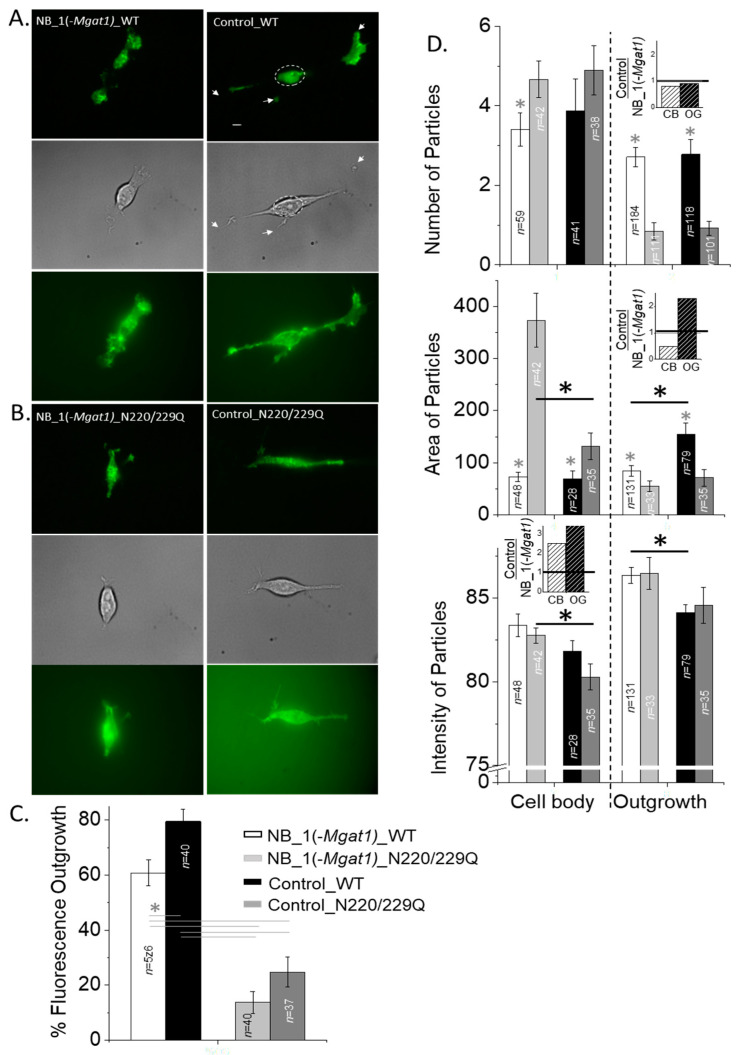
N-Glycosylation processing modified the spatial arrangement of Kv3.1b protein-containing particles in subdomains of the cell membrane. Representative micrographs of glycosylated (WT) (**A**) and unglycosylated (N220/229Q) (**B**) Kv3.1b proteins expressed in NB_1(-Mgat1) (left panels) and control (right panels) cells acquired in TIRF (upper panels), DIC (middle panels) and wide-field (lower panels) modes. Representative scale bar (5 μM) was similar for all acquired images. The dashed circle denotes the cell body and the projections from the cell body represent the outgrowths. (**C**) Percent fluorescence in the outgrowth of parental and N-glycosylation mutant cells lines stably expressing glycosylated (WT) and unglycosylated (N220/229Q) Kv3.1b proteins. Data are presented as the mean ± SEM and were compared by one-way ANOVA (* *p* < 0.03). *n* represents the number of cells analyzed. (**D**) Number (top panel), area (middle panel) and mean intensity (bottom panel) of particles in cell body and outgrowth of NB_1(-Mgat1) and control (NB_1) cell lines expressing glycosylated (WT) and unglycosylated (N220/229Q) Kv3.1b proteins. Insets represent differences in the particle parameters due the type of N-glycan attached to the Kv3.1 protein in cell body and outgrowth. Graphs denote mean ± SEM and were compared by Student’s t-test (*p* < 0.05). (*) denotes a comparison between glycosylated and unglycosylated Kv3.1b in the same cell line and (*) represents a comparison between either glycosylated or unglycosylated Kv3.1b a cell line. *n* denotes the number of particles examined.

Previous studies have shown that the spatial arrangement of Kv3.1b proteins with (Control_WT) and without (Control_N220/229Q) complex type N-glycans were quite different to each other in NB cells [14], similar to those in adult mouse hippocampal-cortical neurons [16]. Subsequently, the aim was to examine whether the type of N-glycan attached to Kv3.1b and/or at the cell surface can alter the distribution of glycosylated and unglycosylated Kv3.1b proteins in subdomains of the cell membranes of NB cell lines. High-contrast images of WT (Figure 5A) and N220/229Q (Figure 5B) Kv3.1b protein tagged with EGFP at the cell surface of live NB_1(-*Mgat1*) (left micrographs) and control (right micrographs) cell lines were obtained by total internal reflection fluorescence (TIRF) microscopy (top panels). Differential interference contrast (DIC) images (middle rows) were acquired in the same plane to designate the clusters of fluorescence signal to the cell body and outgrowths of the cell in TIRF images. Micrographs were also acquired from the same cell after changing the laser beam to obtain wide-field fluorescence excitation (bottom rows). The nucleus is easily observed, and the signal-to-noise ratio was lower in the wide-field images, supporting a clear difference between wide-field and TIRF modes. The spatial arrangement of Kv3.1b proteins at the adherent plasma membrane showed EGFP signal in both subdomains of NB cell lines in the TIRF images. However, there was much more fluorescent signal detected in the outgrowths than cell body for cells expressing WT Kv3.1b protein than the N220/Q protein. It also appeared that WT Kv3.1b expressed in NB_1(-*Mgat1*) cells had less fluorescent signal in the outgrowths relative to the cell body compared to WT Kv3.1b expressed in the NB_1 cell line. These observations were confirmed by quantification of the percent of fluorescence in outgrowth for WT and N220/229Q Kv3.1b proteins expressed in the glycosylation mutant and control cell lines (Figure 5C). 

To further characterize differences due the N-glycan population at the cell surface and the N-glycan type attached to the Kv3.1b protein, we analyzed fluorescence particles for glycosylated (WT) and unglycosylated (N220/229Q) Kv3.1b proteins expressed in the NB_1(-*Mgat1*) and control cell lines. Particle number and area in the cell body and outgrowth were different between glycosylated and unglycosylated Kv3.1b proteins in both parental and glycosylation mutant cell lines (Figure 5D). Further, when comparing either WT or N220/229Q Kv3.1b proteins in the distinct cell lines, particle number was quite similar in outgrowth and cell body. To illustrate the impact of a given N-glycan attached to the Kv3.1b protein had on various particle parameters, the ratio between changes due to complex N-glycans attached to the Kv3.1b protein and those due to oligomannose N-glycans attached to the Kv3.1b protein were plotted (insets). These ratios of the number of particles in the cell body and outgrowth were quite similar, as their ratios were close to 1, indicating there were minimal, if any, changes in particle number due to changing the complex type of N-glycan to oligomannose type (top panel, inset). In contrast, when comparing the N220/229Q Kv3.1b protein expressed in NB_1(-*Mgat1*) to that in the control cell line, particle areas were clearly different in the cell body. A difference in particle area in outgrowths could also be shown between WT Kv3.1b protein expressed in NB_1(-*Mgat1*) and that expressed in control cell line. As such, changes in the particle area due to substituting complex with oligomannose types of N-glycans attached to the Kv3.1b protein in the cell body and outgrowth were quite different (middle panel, inset). Significant differences were not observed for particle intensity between the glycosylated and unglycosylated Kv3.1b proteins in NB_1(-*Mgat1*) or control cell lines. However, differences between unglycosylated Kv3.1b proteins in the cell body of the two distinct cell lines were evident, as were those between glycosylated Kv3.1b proteins in the outgrowths. In both cases, the ratios due to the type of N-glycan attached to Kv3.1b were at least 2-fold greater (bottom panel, inset). These results indicated that the type of N-glycan at the cell surface modifies the number of particles in both domains of NB cells while the effect due to the type of N-glycan attached to the Kv3.1b protein was minimal. In contrast, the type of N-glycan at the cell surface and those attached to the Kv3.1b protein influenced the area and intensity of the fluorescent particles in the cell body and outgrowths of NB cells. 

### 3.5. Opening and Closing Rate of Outward Ionic Current Decreased as N-Glycan Branching Is Lessened

**Figure 6:**
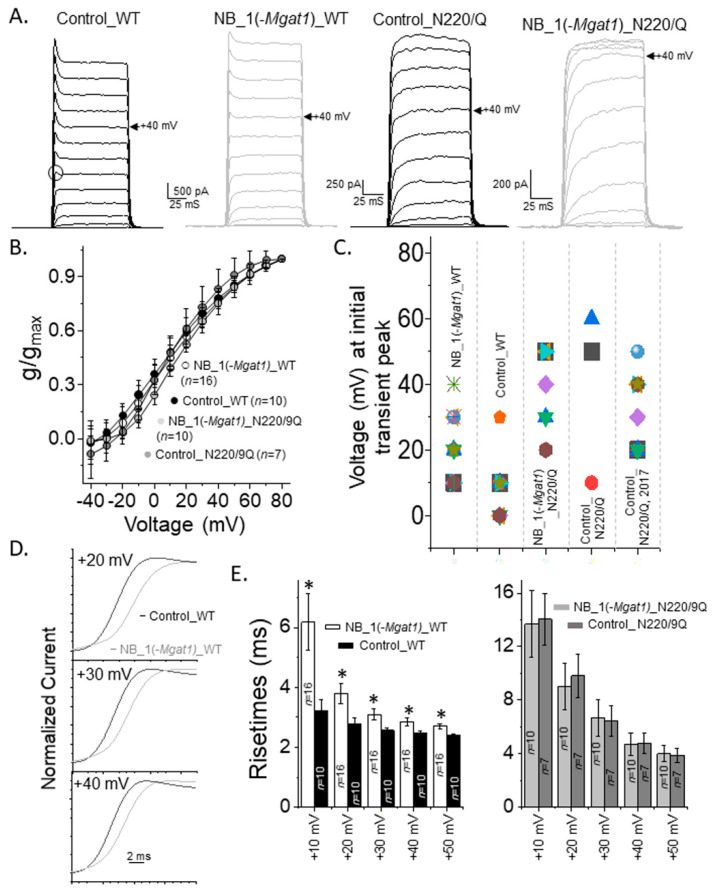
Opening rate of outward ionic current decreased as N-glycan branching is lessened. (**A**) Activation currents were elicited by holding cells at −50 mV and stepping from −40 mV to +80 mV in 10 mV increments for 100 ms. Whole cell currents were acquired for glycosylated Kv3.1b expressed in parental (Control_WT) and glycosylated mutant (NB_1(*-Mgat1*)_WT) cells and unglycosylated Kv3.1 in the mutant cell line (NB_1(*-Mgat1*)_N220/229Q). Open circle denotes transient peak at +10 mV. Control cell line is the parental cell lines, NB_1. Arrow points to current recorded at +40 mV. (**B**) Conductance-voltage (g/gmax) curves obtained from non-inactivating currents with and without transient peaks expressed by glycosylated (WT) and unglycosylated (N220/229Q) Kv3.1b in NB_1 and NB_1(-*Mgat1*). (**C**) Scatter plot illustrating voltage at which the initial transient peak was observed from non-inactivating current with transient peaks of the various cell lines and those from our past reports [16]. (**D**) Whole cell currents from activation voltage protocols in (**A**) were expanded for Control_WT and NB_1(*-Mgat1*)_WT cells lines. (**E**) Rise times of activation currents of the WT Kv3.1 (left panel) and N220/229Q (right panel) expressing cell lines. Data are presented as the mean ± SEM and were compared by Student’s t-test (* *p* < 0.05).

Occupancy of the N-glycosylation sites of Kv3.1b expressing cells reduced the gating kinetics of Kv3 channels in NB cells [13]. To demonstrate that substitution of complex-type N-glycans with oligomannose type can modify Kv3 channel activity, whole cell recordings were obtained from NB_1 (control) and NB_1(-*Mgat1*) cells stably expressing glycosylated (WT) or unglycosylated (N220/229Q) Kv3.1b proteins (Figure 6A). The predominant type of current expressed was non-inactivating with transient peaks for glycosylated Kv3.1b in parental (Control_WT) and N-glycosylation mutant (NB_1(-*Mgat1*)_WT) cell lines, while this current type was less than the majority for unglycosylated Kv3.1b in mutant and parental cell lines (Table 1). The transient peak represents the highest point of current that readily decreases to a steady state immediately following a depolarization step. Non-inactivating without transient peaks (Control_N220Q) or inactivating current (NB_1(-*Mgat1*)_N220/229Q) types were more common for cell lines stably expressing unglycosylated Kv3.1b protein. Inactivating current types have currents that saturate at higher depolarization test potentials. In all cases, the voltage dependence of activation for non-inactivating currents expressed by the Kv3 channels containing either glycosylated and unglycosylated Kv3.1b proteins in the parental or glycosylation mutant cell lines were similar as currents were detected when the applied test potential was greater than −20 mV (Figure 6B). A further 50% of Kv3 channels were activated at a similar test potential (test potential at which g/g_max_ = 0.5 (V_0.5_) was 9.0 ± 1.5 mV, *n* = 16, NB_1(-*Mgat1*)_WT; 7.3 ± 1.0 mV, *n* = 10; Control_WT; 14.4 ± 1.7 mV, *n* = 10; NB_1(-*Mgat1*)_N220/229Q; 9.0 ± 1.1 mV, *n* = 7, Control_N220/229Q), and the level that activated channels climbed with increased test potential (dV was 24.5 ± 2.3 mV, NB_1(−*Mgat1*)_WT; 24.3 ± 1.5 mV, Control_WT; 20.7 ± 2.2 mV, NB_1(-*Mgat1*)_N220/229Q; 18.3 ± 1.3 mV, Control_N220/229Q) was similar to that determined by fitting the data points with a Boltzmann isotherm.

To further assess differences in the non-inactivating currents with transient peaks, we correlated the initial occurrence of transient peaks with depolarizing test potentials (Figure 6C). The selected transient peak for an activation current recording is denoted by the open circle for control_WT in panel A. The lowest voltage at which transient peaks were detected from currents elicited by the activation voltage protocol were at higher test potentials for NB_1(-*Mgat1*)_WT than those for Control_WT, while those for both Control_DM and NB_1(-*Mgat1*)_DM cell lines were even higher. Since only three Control_N220/229Q cells expressed non-inactivating currents with transient peaks, we analyzed non-inactivating currents recorded from the same cell line at an earlier date [16]. The voltage for detecting initial transient peaks were quite reproducible for NB cell lines expressing the N220/229Q Kv3.1b protein.

**Figure 7:**
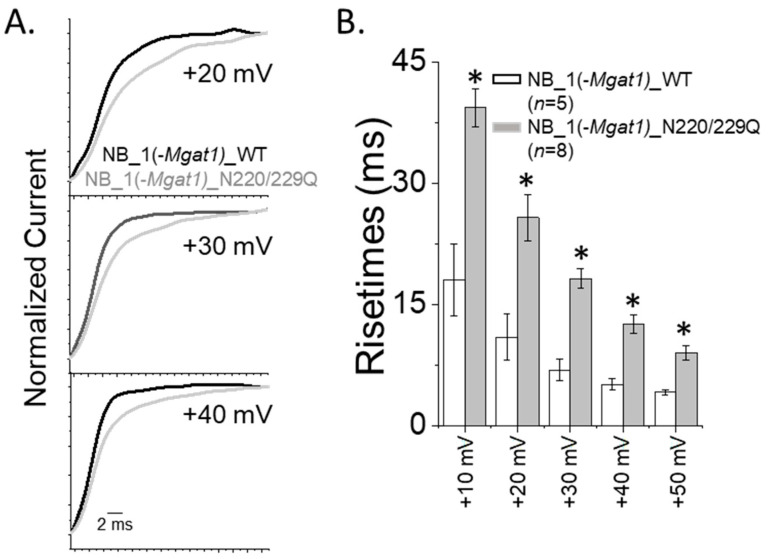
Delayed opening rates of inactivating currents with altered N-glycan branching. (**A**) Whole cell currents of inactivating current were expanded for NB_1(*-Mgat1*)_WT and NB_1(*-Mgat1*)_DM cells lines. (**B**) Rise times obtained from inactivating currents of NB_1(*-Mgat1*)_WT and NB_1(*-Mgat1*)_DM expressing cells. Data are presented as the mean ± SEM and were compared by Student’s t-test (* *p* < 0.05).

Since past studies have shown that the opening of the Kv3 channels containing glycosylated Kv3.1b protein were much faster than those containing its unglycosylated counterpart [13,35], we examined the opening rates of the outward ionic currents generated by cell lines with differences in N-glycosylation processing of Kv3.1b. Expansion of the time scale for non-inactivating current type expressed by glycosylated Kv3.1b with complex type (Control_WT) and those with oligomannose type (NB_1(-*Mgat1*)_WT) showed that currents rise to their peak currents slower when the glycans were less processed (Figure 6D). Slower mean rise times were shown at these voltages, along with those at additional applied voltages for the glycosylated Kv3.1 protein with oligomannose type compared to that with complex type N-glycans (Figure 6E). In both cases, rise times observed for the Kv3 channel containing unglycosylated Kv3.1b protein were quite similar, but much slower than their glycosylated counterparts. Rise times were also examined for inactivating currents of Kv3.1b expressed in the glycosylation mutant cell line. Enlargement of the inactivating currents at +20 mV, +30 mV and +40 mV for NB_1(-*Mgat1*)_WT and NB_1(-*Mgat1*)_N220/229Q cell lines illustrated a more rapid opening for Kv3 channels containing glycosylated Kv3.1b than those with unglycosylated Kv3.1b (Figure 7A). Further quantification of the rise times of outward ionic currents from NB_1(-*Mgat1*)_WT and NB_1(-*Mgat1*)_N220/229Q cell lines verified that N-glycans attached to the Kv3.1b protein increased opening rates (Figure 7B). 

**Figure 8:**
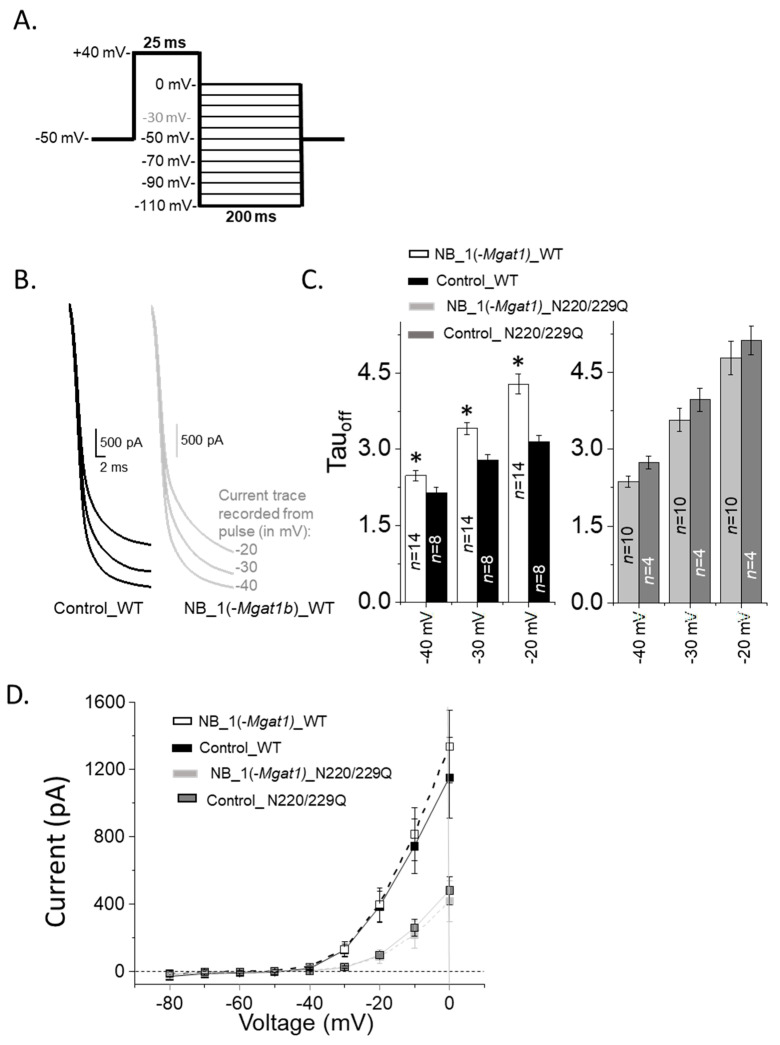
Closing rate of outward ionic current decreased as N-Glycan branching is lessened. (**A**) A deactivation protocol (**left panel**) was used to elicit whole currents (**right panel**) in NB cells stably expressing various forms of the Kv3.1b protein. (**B**) Expansion of deactivation currents at −40, −30 and −20 mV for glycosylated Kv3.1b expressed in parental (Control_WT) and glycosylated mutant (NB_1(*-Mgat1*)_WT) cells. (**C**) Deactivation time constants (Tau_off_) of deactivation currents from WT Kv3.1 (left panel) and N220/229Q (right panel) expressing cell lines. (**D**) Current–voltage curves were obtained from the tail currents of the deactivation protocol for glycosylated (WT) and unglycosylated (N220/229Q) Kv3.1 α-subunits expressed in NB_1 and NB_1(-*Mgat1*). Data are presented as the mean ± SEM and were compared by Student’s *t*-test (* *p* < 0.05).

Next, our aim was to assess changes in the closing rate of the outward ionic currents in NB cells that stably expressed Kv3.1b proteins. The closing rates were determined by increasing the outward ionic current with a depolarization step (+40 mV), and subsequently applying hyperpolarizing steps, resulting in a rapid decay of the ionic current or closing of Kv3 channels (Figure 8A). Enlargement of the time scale of deactivating currents from parental and N-glycosylation mutant cell lines expressing WT Kv3.1b revealed clear differences in the closing rates of the currents at three test potential (−40 mV, −30 mV and −20 mV) (Figure 8B). Differences in closing rates were shown by the mean deactivation rate constants (Tau_off_) which involves fitting the current curves at the various test potentials (Figure 8C). The closing rates for the NB_1(-*Mgat1b*)_WT cell line were markedly slowed relative to the Control_WT cell line, while they were somewhat similar to the NB_1(-*Mgat1b*)_N220/229Q and Control_N220/229Q cell lines. 

Since the opening and closing rates were slowed when N-glycans were shortened and fewer, we measured the magnitude of the tail currents from the deactivation voltage protocol (Figure 8D). The magnitude of the tail currents for NB_1(-*Mgat1b*)_WT cell line were quite similar to the Control_WT cell line but were larger than the cell lines expressing N220/229Q Kv3.1. In all cases, positive currents were observed at potentials greater than −50 mV, while zero to minimal negative current amplitudes were detected at more negative potentials than −50 mV. These results suggest that the number of open Kv3.1b channels were greater for cell lines expressing WT Kv3.1b than those expressing N220/229Q Kv3.1b while the driving force on K^+^ ions was similar for all cell lines. 

## 4. Discussion

This study demonstrated that aberrant changes in N-glycosylation processing of the Kv3.1b α-subunit impaired motor activity in zebrafish larvae. Spinal cord CaP motor neurons were shown to express Kv3.1b, and CaP neurons expressing fully processed Kv3.1b glycoprotein developed much like those expressing EGFP. However, CaP neurons were markedly aberrant when expressing partially glycosylated Kv3.1 protein. A GnT-I knockdown was engineered to show that alterations in N-glycan type could impact Kv3 channel function. Activation and deactivation kinetics of Kv3 channels containing Kv3.1b were altered by occupying N-glycosylation sites with different N-glycans types in NB cells. Dispersal of Kv3.1b to outgrowths relative to soma were enhanced by occupied N-glycosylation sites and more processed N-glycans. These in vivo and in vitro studies corroborate our proposed model that N-glycosylation processing of Kv3 channels containing Kv3.1b is critical in neuronal development and function of vertebrate.

Past studies reported expression of Kv3.3 channels in CaP neurons as early as 24 hpf and increases in expression in the next 24 h, correlating with CaP neuronal development in zebrafish [10,25]. Further Kv3 channels containing Kv3.1 are vital components in setting the firing pattern of interneurons from rat spinal cord [36]. Co-localization of Kv3.1b with Znp-1 indicated that Kv3.1b is present in the spinal cord of CaP neurons. Various combinations of Kv3 α-subunits form homomeric and heteromeric Kv3 channels, and Kv3.1 and Kv3.3 combine to form Kv3 channels in neurons of the medial nucleus of trapezoid body [37,38]. Kv3.1 and Kv3.3 α-subunits have their N-glycosylation sites occupied by complex type N-glycans in rat spinal cord [9]. Taken together, it is likely that Kv3 channels in CaP neurons of zebrafish exist as heteromeric complexes of Kv3.1b and Kv3.3a α-subunits with occupied N-glycosylation sites.

To date, we showed that occupancy of one or both N-glycosylation sites of Kv3.1b [9,33] impacts its plasma membrane localization, and Kv3 channel opening and closing rates in cultured cell lines [13,15,35], including primary neurons [16]. Our in vivo study revealed that vacancy of the N220 N-glycosylation site of Kv3.1b disrupted CaP neuron development. The average size of CaP neurons was reduced by ~3-fold compared to CaP neurons expressing WT Kv3.1b or EGFP which correlated with decreases in branching and synapse formation. Further, CaP neurons expressing Kv3.1b with both sites vacant had >90% of severely deformed and maldeveloped embryos. Spontaneous motor activity in larvae expressing N220Q Kv3.1b motor neurons was impaired. The small reduction in motor activity was expected since recombinant pTol vector gives a mosaic expression pattern and only a few to several CaP neurons are affected. For example, out of the 11 embryos imaged for N220Q expression (Figure 1), the number of CaP neurons expressing N220Q from an embryo did not exceed 5–10. Previously, the importance of Kv3.1 in coordinated motor skills was demonstrated in Kv3.1 knock out mice [39]. Thus, it appears that CaP neuron development requires occupancy of both N-glycosylation sites of Kv3.1b.

Non-conducting and conducting functions of Kv3.1b may impede the development of CaP neurons expressing N220Q Kv3.1b. Non-conducting functions of ion channels include roles in cell signaling, cell adhesion and cytoskeleton network [40]. Ecotopic expression of glycosylated Kv3.1b proteins in NB cells increased their migratory rates [13]. Moreover, rates were slowed when an N-glycosylation site was vacant and considerably slowed with both sites vacant relative to fully glycosylated Kv3.1b [13]. Our images revealed that axons crossed the horizontal myoseptum, but barely entered the ventral myotome without branches. Soma without neurites were also observed, but which motor neuron was unclear. Our results suggest that axonal outgrowths follow the path established by pre-existing acetylcholine receptors clusters [41,42] but fail to result in branching. Since spiking activity is absent for initial outgrowth formation of CaP axons [41,42,43], it may be that non-conducting functions of Kv3.1b contribute to this process. Kv3 channels are different from other Kv channels in that their activation curves are shifted to more depolarized potentials and they have very rapid activation and deactivation rates [37]. Previously, we showed Kv3 channels expressing the partially glycosylated Kv3.1b have reduced opening and closing rates [13,35]. Changes in Kv3 channel gating were observed for the infant-onset mutation of spinocerebellar ataxia in Kv3.3 [11], and subsequently altered firing rates in Kv3.3-expressing CaP neurons and developing Purkinje cells of zebrafish [10,44]. Shortened growth and limited branching of axons could result from decreased firing rates of Kv3 channels containing partially glycosylated Kv3.1b in CaP neurons. Taken together, it is arguable that maldevelopment of N220Q Kv3.1b-expressing CaP neurons is due to both non-conducting and conducting functions of Kv3 channels.

Maldevelopment of N220Q Kv3.1b-expressing CaP neurons may result from aggregation of N220Q in the endoplasmic reticulum (ER). A role of N-glycans attached to proteins is quality control of protein folding which occurs in the ER [45]. Although this process is vital for production of viable and healthy cells, over accumulation of misfolded proteins in the ER may result in ER stress, and lead to neuronal cell death [46]. Whole-cell current measurements showed current densities of WT, N220Q, N229Q, and N220/229Q Kv3.1b stably expressed in NB cells were not significantly different, indicating that all Kv3.1b forms were expressed in plasma membrane [13]. Nonetheless, the trend was less Kv3.1b protein in the plasma membrane as N-glycosylation sites were reduced. Taken together, retention of partially glycosylated Kv3.1b in the ER could cause ER stress and result in maldevelopment of N220Q Kv3.1b-expressing CaP neurons. Severe malformation of microinjected N220/229Q Kv3.1b embryos could also be supported by ER stress.

Kv3.1b localization in the outgrowth relative to cell body was higher for Kv3.1b with oligomannose N-glycans than without N-glycans, supporting previous report comparing Kv3.1b with and without complex N-glycans [15]. It was also observed that substitution of complex with oligomannose N-glycans reduced Kv3.1b levels delivered to outgrowths by ~5% while substitution of hybrid N-glycans caused about a 15% reduction [16]. As such, less processed N-glycans yields less Kv3.1b glycoprotein localization to outgrowths versus cell body. The number, size, and intensity of Kv3.1b-containing particles distributed between the cell body and outgrowth were dependent on N-glycan type at the cell surface. Size and intensity were also dependent on N-glycan type attached to Kv3.1b. Replacement of complex N-glycans with hybrid N-glycans also showed differences in Kv3.1b particle distribution [16]. Since firing patterns are dependent on the spatial arrangement of Kv channels expressed in neurons [47], it is probable that N-glycan type of Kv3.1b and those of the plasma membrane in which Kv3.1b is embedded can control Kv3.1b multicomplexes in the plasma membrane and thereby action potential-spiking of CaP neurons.

Kv3 channels are critical in regulating electrical firing patterns in fast-spiking neurons. [7]. Kv3 channels containing glycosylated Kv3.1b have faster activation rates than those channels with unglycosylated Kv3.1b [13,35]. Non-inactivating currents with transient peaks enhance activation rates relative to other current types. Kv3 channels containing glycosylated Kv3.1b express this type of current more often than those with unglycosylated Kv3.1b [13]. Herein, most of the whole cell currents expressed by Kv3 channels containing Kv3.1b with oligomannose N-glycans were non-inactivating with transient but the transient peak occurred at higher depolarized potentials than when these channels contained Kv3.1b with complex N-glycans. Activation and deactivation rates were slowed in Kv3 channels containing glycosylated Kv3.1b with oligomannose N-glycans versus complex N-glycans. Moreover, voltage midpoints of channel activation were alike, and tail currents approached zero at similar membrane potentials. Opening and closing rates of Kv3 channels containing Kv3.1b with hybrid N-glycans were also slowed compared to those channels with complex N-glycans [16]. These results indicate N-glycans of Kv3.1b stabilize the opening and closing states of Kv3 channels. Stabilization of these states could be due to changes in protein structure or decreased protein dynamics. A study reported that N-glycosylation of proteins likely favors protein stability by reducing intrinsic dynamic properties [48]. Since more processed N-glycans could enhance the opening and closing states to a greater degree, it suggests that as N-glycans become larger and more hydrophilic, the protein dynamics decrease, thereby allowing Kv3 channels to rapidly open and close. Thus, the results indicate that N-glycan types of Kv3.1b can modulate the opening and closing rates of Kv3 channels, suggesting N-glycosylation processing of Kv3.1b controls the duration of a nerve impulse.

## 5. Conclusions

In conclusion, in vivo studies verify the importance of occupied N-glycosylation sites of Kv3.1b and support that changes in Kv3.1b spatial arrangement and gating properties identified in cell culture can be extrapolated to fast-spiking neurons. Current in vitro studies suggest that higher processing of N-glycans of Kv3.1b stabilize the opening and closing states of Kv3 channels by reducing intrinsic dynamics of the protein. N-Glycans at the cell surface and those attached to Kv3.1 could modify the localization of Kv3.1b containing multicomplexes. Our results provide novel insight in which aberrant changes in the N-glycosylation pathway alter Kv3 channel localization and activity, resulting in maldevelopment of motor neurons, and defective motor activity. Further our findings provide fundamental knowledge to better understand the neurological component of CDG, and other neurodegenerative diseases, suggesting potential drug targets for therapeutic interventions in human neurodegenerative diseases.

## Figures and Tables

**Table 1:** Type of current expressed by N-glycosylation mutant and parental cell lines.

Cell Line	Non-Inactivating w/Transient Peaks	Non-Inactivating w/o Transient Peaks	Inactivating
NB_1(-*Mgat1*)_WT	70%	13%	17%
Control_WT	91%	0%	9%
NB_1(-*Mgat1*)_N220/229Q	47%	12%	41%
Control_N220/229Q	30%	40%	30%

## Data Availability

Data not available in publications, can be obtained from R.A.S. upon request.

## Supplementary Materials

The following are available online at https://www.mdpi.com/article/10.3390/biology10060486/s1, Multimedia, Figure S1. EGFP expressed in a CaP neuron (file name: EGFPonly_inVivo_ani06.mp4), Figure S2. WT Kv3.1b tagged with EGFP expressed in a CaP neuron (file name: WTKv3.1_inVivo_ani09.mp4), Figure S3. N220Q Kv3.1b tagged with EGFP expressed in a CaP neuron (file name: 220mu_inVivo_ani01148hpf_Kv3.mp4), Figure S4. Co-expression of Znp-1 and EGFP in a CaP neuron (file name: EGFPonly_IHC_ani07.mp4), Figure S5. Co-expression of Znp-1 and N220Q Kv3.1b tagged with EGFP in a CaP neuron, and an adjacent CaP neuron expressing Znp-1 but not N220Q Kv3.1b tagged with EGFP (file name: 220Kv3.1Mu_IHC_ani011Kv3.mp4), Figure S6. western blot of Figure 4A, Figure S7. western blot of Figure 4B, Figure S8: western blot of Figure 4C.
