# Supplementary figures and images for: Compromised N-Glycosylation Processing of Kv3.1b Correlates with Perturbed Motor Neuron Structure and Locomotor Activity
Source: Biology (Basel). 2021 May 30;10(6):486. doi: 10.3390/biology10060486 (PMC8229559; doi:10.3390/biology10060486)

Figure S-6

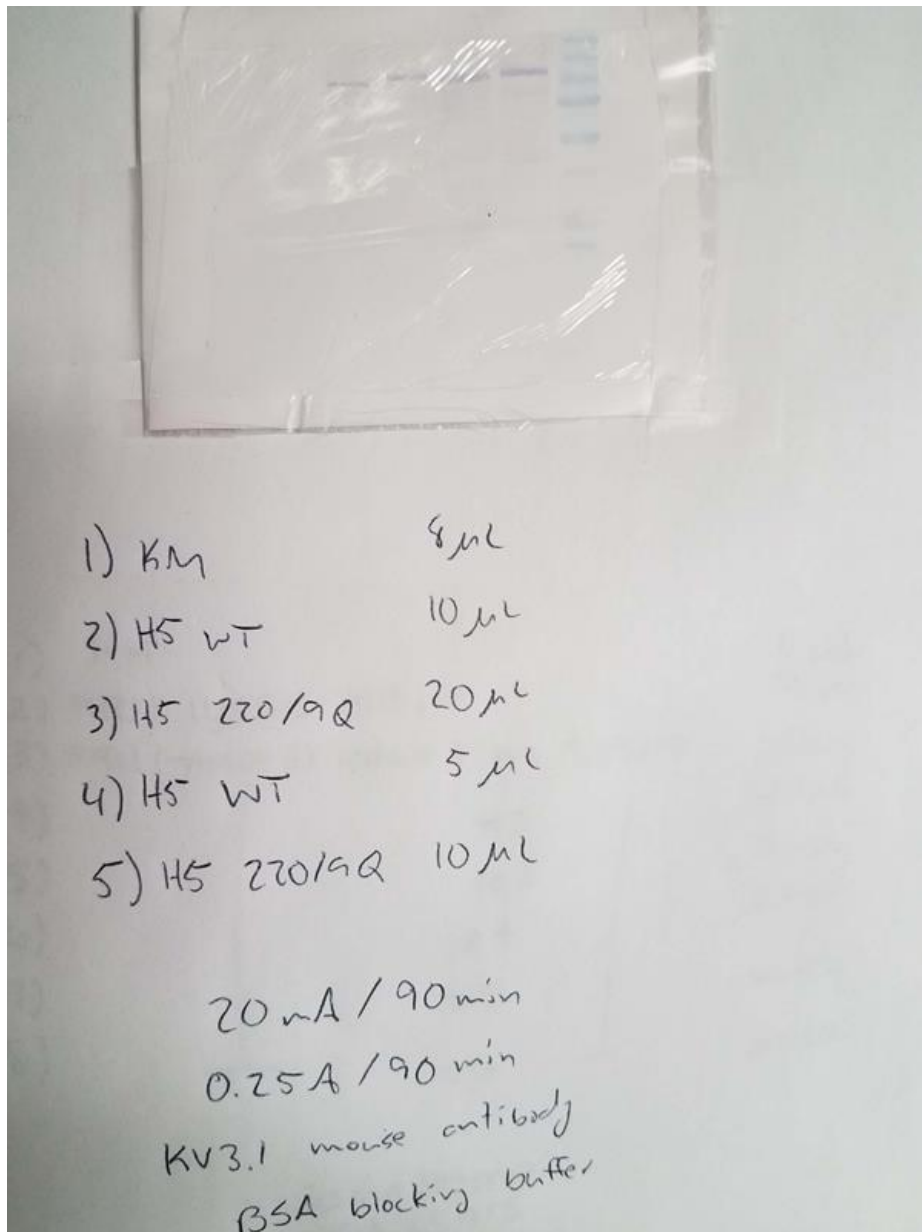

Figure S-7

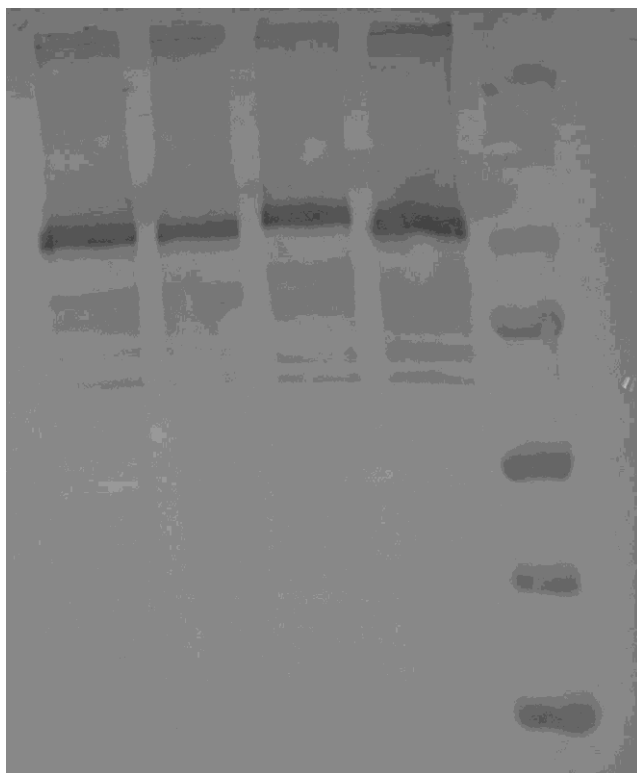

Figure S-8

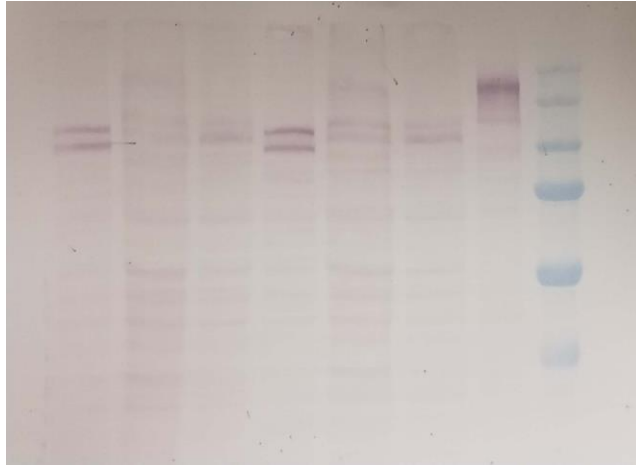

Supplement: Supplementary file 1 [file biology-10-00486-s001.zip › S6,7,8_Western Blots_images.pdf]
